# Association between Antiepileptic Drugs and Incident Parkinson’s Disease among Patients Followed in German Primary Care Practices

**DOI:** 10.3390/brainsci13030450

**Published:** 2023-03-06

**Authors:** Karel Kostev, Corinna Doege, Louis Jacob, Lee Smith, Ai Koyanagi, Celina Gollop, Anette Schrag

**Affiliations:** 1 University Clinic, Philipps University, 35043 Marburg, Germany; 2 Epidemiology, IQVIA, 60549 Frankfurt, Germany; 3 Department of Pediatric Neurology, Center for Pediatrics and Adolescent Medicine, Central Hospital, 28211 Bremen, Germany; 4 Research and Development Unit, Parc Sanitari Sant Joan de Déu, CIBERSAM, ISCIII, 42, Sant Boi de Llobregat, 08830 Barcelona, Spain; 5 Department of Physical Medicine and Rehabilitation, Lariboisière-Fernand Widal Hospital, AP-HP, Paris Cité University, 75010 Paris, France; 6 Centre for Health, Performance and Wellbeing, Anglia Ruskin University, Cambridge CB1 1PT, UK; 7 Institució Catalana de Recerca i Estudis Avançats (ICREA), Pg. Lluis Companys 23, 08830 Barcelona, Spain; 8 Neurology, University College London, London WC1N 3BG, UK

**Keywords:** antiepileptic drug, epilepsy, Parkinson’s disease, Germany

## Abstract

Background: The aim of this study was to analyze whether prescriptions of antiepileptic drugs (AEDs) are significantly associated with an increased incidence of Parkinson’s disease (PD) in the German population. Methods: This study used data from German primary care practices found in the Disease Analyzer database (IQVIA) and included all patients aged ≥18 years who were diagnosed with PD between January 2010 and December 2021 (index date). The controls were patients without PD matched (1:1) by age, sex, and pre-diagnostic observation time in years. Associations between AED prescriptions (any AED as well as separate evaluations for carbamazepine, lamotrigine, levetiracetam, sodium valproate, gabapentin, and pregabalin) and subsequent diagnosis of PD were examined using a logistic regression model adjusted for epilepsy, restless legs syndrome, and neuropathy diagnoses. Results: We identified 24,950 cases that were matched with 24,950 controls (mean age 75.2 years, 47.3% women). Diagnoses of epilepsy, restless legs syndrome, and neuropathy as well as AED prescription were significantly associated with an increased incidence of PD. In the multivariate analysis, incidence of PD was significantly associated with epilepsy (OR: 1.91; 95% CI: 1.69–2.15), restless legs syndrome (OR: 3.02; 95% CI: 2.73–3.34), and neuropathy (OR: 1.53; 95% CI: 1.44–1.62)), as well as the prescription of any AED (OR: 1.43; 95% CI: 1.33–1.53), sodium valproate (OR: 2.39; 95% CI: 1.84–3.11), gabapentin (OR: 1.36; 95% CI: 1.22–1.52), and pregabalin (OR: 1.28; 95% CI: 1.15–1.41)**.** Conclusion: Prescriptions of AEDs, including sodium valproate, gabapentin, and pregabalin, were associated with an increased risk of subsequent PD, even after adjustment for underlying diagnoses. Further studies are needed to confirm the present results.

## 1. Introduction

Parkinson’s disease (PD) is a neurodegenerative disorder with a prevalence of 1–2% among people aged over 60 years that increases with age; consequently, the prevalence estimations of PD may be affected by the under-diagnosis of PD among the most elderly people [1]. The incidence of PD has been increasing over the last few decades, rendering PD the fastest-growing neurological disorder worldwide [2]. The investigation of risk factors for PD has become an important research topic in recent years. For example, epilepsy has been found to be associated with a higher risk of subsequent PD onset [3,4]. Associations between different risk factors and pre-diagnostic presentations of PD have been reported for chronic diseases such as diabetes mellitus, hypertension, and depression, with a significant association found for epilepsy (Odds Ratio (OR), 2.50; 95% Confidence Intervals (CI), 1.63–3.83) [4]. Furthermore, some non-motor features of PD can predate the diagnosis of PD by several years.

Although the association between prescription medications and subsequent diagnosis of PD is of particular clinical relevance, it has not been comprehensively studied. Recently, Belete and colleagues reported that prescriptions of several antiepileptic drugs (AED) are significantly associated with an increased incidence of PD in the UK [5]. However, the association differed depending on the type of AED, being strongest for valproate (OR: 3.82; 95% CI: 2.41–6.05) and weakest (and statistically non-significant) for carbamazepine (OR: 1.43; 95% CI: 0.97–2.11). The authors also conducted analyses in a hospital setting and studied the four most commonly prescribed AEDs in the UK, but these findings may not be generalizable to other AEDs or other countries [5]. Therefore, we examined whether these associations can be replicated in German primary care practices.

## 2. Methods

### 2.1. Database

This study used data from German primary care practices found in the Disease Analyzer database (IQVIA). Details of the methodology behind this database have been published previously [6]. In brief, the Disease Analyzer database contains data on demographic variables, diagnoses, and prescriptions from general and specialized practices in Germany. 

The quality of the data is assessed every month based on several criteria (e.g., completeness of documentation and linkage between diagnoses and prescriptions). Practices included in the database are selected based on the yearly statistics published by the German Medical Association, which include information on physician’s age, specialty group, community size category, and German federal state. Prior research has shown that the Disease Analyzer database is representative of all practices in Germany [7].

German law allows for the use of anonymous electronic medical records for research purposes under certain conditions. According to this legislation, it is not necessary to obtain informed consent from patients or approval from a medical ethics committee for this type of observational study, which contains no directly identifiable data. Therefore, no waiver of ethical approval was obtained from an Institutional Review Board (IRB) or ethics committee. The authors had no access to any identifying information at any time during the analysis of the data.

### 2.2. Study Population

The study population included all patients aged ≥18 years with a diagnosis of PD (ICD-10 code: G20) made between January 2010 and December 2021 (index date) who had been registered in the database for at least one year prior to the index date. This latter inclusion criterion was chosen to ensure that no patients recently registered in the database who had an existing diagnosis of PD were included. 

**Figure 1:**
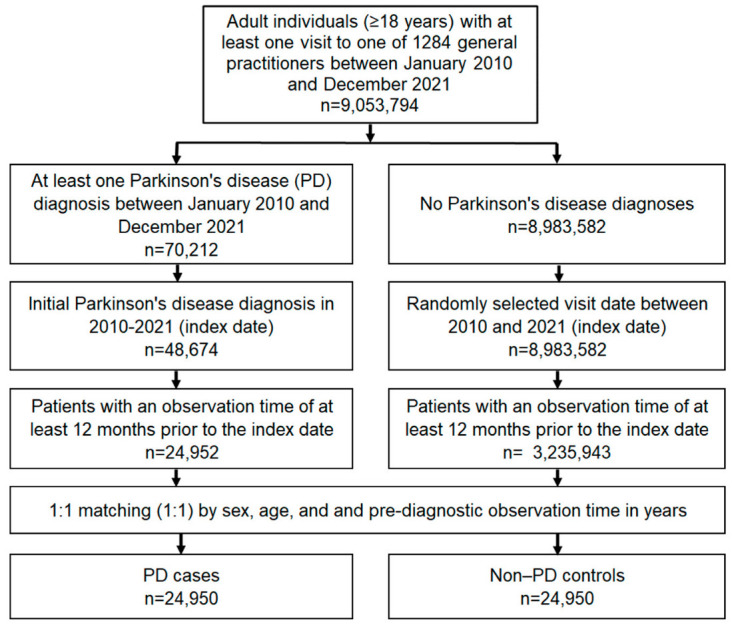
Flow diagram concerning study participants.

Controls were patients without PD who were matched (1:1) by age, sex, and their pre-diagnostic observation time in years. For individuals without PD, the index date was a randomly selected visit date between January 2010 and December 2021. A flow diagram of study participants is shown in Figure 1.

### 2.3. Statistical Analyses

Demographic and clinical characteristics of cases and controls after 1:1 propensity-score matching were evaluated using the Wilcoxon signed-rank test for continuous variables, the McNemar test for categorical variables with two categories, and the Stuart–Maxwell test for categorical variables with more than two categories. Associations between AED prescriptions (any AED as well as separate analyses for carbamazepine, lamotrigine, levetiracetam, sodium valproate, gabapentin, and pregabalin) and subsequent diagnosis of PD (dependent variable) were examined using logistic regression. As antiepileptic medications can be prescribed for other conditions that may themselves be associated with subsequent diagnosis of PD, such as epilepsy, restless legs syndrome, and painful diabetic or toxic and drug-induced neuropathy, we also examined the association between the conditions and these diagnoses. ICD-10 codes were used for epilepsy (ICD-10: G40), restless legs syndrome (ICD-10: G25.8), and diabetic/toxic/drug-induced neuropathy (ICD-10: G62, E10.4, E11.4, E14.4) diagnoses. Multivariate logistic regression models included all AEDs examined and the diagnoses listed above. 

We performed sensitivity analyses including only AEDs prescribed at least 12 months before the index date or at least 36 months before the index date. *p*-values < 0.01 were considered statistically significant. 

## 3. Results

We identified 24,950 cases, which were matched with 24,950 controls. The mean (standard deviation, SD) age on the index date was 75.2 (10.5) years and 47.3% of patients were female. The majority of patients were aged between 71–80 (41.7%) or >80 (33.3%), with just 9.3% aged ≤60 years. On average, both the cases and controls had a pre-observation time of 7.8 (SD 5.0) years prior to the index date (Table 1). 

In the univariable regression analysis, epilepsy (OR: 2.39; 95% CI: 1.14–2.67, *p* < 0.001), restless legs syndrome (OR: 3.62; 95% CI: 3.28–4.00, *p* < 0.001), and drug-induced/toxic/diabetic neuropathy (OR: 1.84; 95% CI: 1.74–1.95, *p* < 0.001) were all associated with subsequent diagnosis of PD. Each AED was also significantly associated with subsequent PD. In the multivariable regression analysis, which was adjusted for epilepsy, restless legs syndrome, and neuropathy, the prescription of any AED was significantly associated with an increased incidence of PD (OR: 1.43; 95% CI: 1.33–1.53, *p* < 0.001). Some 12.1% of the PD cases versus 6.5% of the controls had received at least one AED prescription. The strongest association was seen for sodium valproate (OR: 2.39; 95% CI: 1.84–3.11, *p* < 0.001), followed by gabapentin (OR: 1.36; 95% CI: 1.22–1.52, *p* < 0.001) and pregabalin (OR: 1.28; 95% CI: 1.15–1.41, *p* < 0.001) (Table 2). 

However, after adjustment for the diagnoses specified above and other AEDs, there was no longer any association between lamotrigine, carbamazepine, and levetiracetam and PD. Epilepsy diagnosis (OR: 1.91; 95% CI: 1.69–2.15, *p* < 0.001), restless legs syndrome (OR: 3.02; 95% CI: 2.73–3.34, *p* < 0.001), and neuropathy (OR: 1.53; 95% CI: 1.44–1.62, *p* < 0.001) remained associated with PD after adjustment for AEDs (Table 2).

The sensitivity analyses showed that the adjusted associations between gabapentin (OR: 1.40; 95% CI: 1.24–1.60, when prescribed at least 12 months before the index date, and OR: 1.41; 95% CI: 1.14–1.74, when prescribed at least 36 months before the index date), pregabalin (OR: 1.49; 95% CI: 1.33–1.67, when prescribed at least 12 months prior to the index date, and OR: 1.98; 95% CI: 1.54–1.2.53, when prescribed at least 36 months prior to the index date) and PD were confirmed in both sensitivity analyses. The odds ratio for sodium valproate was still significantly increased when the drug was prescribed 12 months prior to the index date or earlier (OR: 1.24; 95% CI: 1.60–2.87); however, the adjusted OR was no longer significant (OR: 1.11; 95% CI: 0.70–1.75, *p* = 0.663) when sodium valproate was prescribed 36 months prior to the index date or earlier (Table 3).

## 4. Discussion

In this case control study, prescriptions of all AEDs investigated were associated with a subsequent diagnosis of PD. In the multivariable regression analyses, even when adjusting for underlying diagnoses of epilepsy, restless legs syndrome, and neuropathic disorders, sodium valproate, gabapentin, and pregabalin were significantly and positively associated with incident PD. Although the association between each AED and subsequent PD became weaker after adjustment for the diagnoses these AEDs were prescribed for, the associations were still strong and significant. 

Overall, our results confirm the findings of Belete et al. [5], with similar ORs for any AED, sodium valproate, and carbamazepine in both studies. Although Belete et al. also found significant positive associations for lamotrigine and levetiracetam, we did not find a significant association for either drug in the multivariate analysis. This is likely due to an additional adjustment for underlying diagnoses, which we found to be independently associated with incident PD. Furthermore, our study found that gabapentin and pregabalin, which were not examined in the previous study, were also associated with the onset of PD, even after adjustment. Whilst the studies differed in terms of AED prescription rates, which were 4.3% in the PD cases in the study of Belete et al. and 12.1% in our study, the results of our study confirm and expand those of Belete et al. in a German collective. Whilst we cannot rule out the possibility that the diagnoses for which AEDs are prescribed may differ in Germany and the UK, or at least that the proportions of AED prescriptions differ in both countries, the results of both studies are broadly similar [8]. In addition, we have expanded the findings by adding new associations for further AEDs and examining the differential contribution of underlying diagnoses and AED prescription.

While it is well known that parkinsonism is induced by sodium valproate, it is less commonly described as an adverse effect of other AEDs [9,10]. Pacheco-Paez et al. reported a significant association between gabapentin and pregabalin and parkinsonism: among 5,653,547 case safety reports from the World Health Organization individual case safety report database, 4925 parkinsonism reports were found for patients taking pregabalin and 4881 for those receiving gabapentin [11]. In rare cases, parkinsonism cases were also reported in patients treated with lamotrigine [12]. No parkinsonism cases have yet been reported among patients treated with levetiracetam [13]. Indeed, this drug can also be used for the management of levodopa-induced dyskinesias in patients with Parkinson’s disease [14]. Levetiracetam was also the only AED that was not associated with PD in the multivariate analysis in our study. Notably, our sensitivity analysis suggested that whilst the association between gabapentin and pregabalin and incident PD may be a long-term side effect, parkinsonism associated with sodium valproate is a short-term side effect since prescriptions issued in the recent past but not in the longer term were associated with subsequent PD.

In our study, epilepsy, restless legs syndrome, and neuropathy were each independently associated with PD. Associations between epilepsy and PD [3,4] and between restless legs syndrome and PD [15,16] have already been reported in the past. The prevalence of restless legs syndrome is increased in PD patients compared to the general population, and both epilepsy and restless legs syndrome have been reported as an early clinical feature of PD [16,17]. Furthermore, pain is a common non-motor symptom of PD, and neuropathy is increased in PD patients, including in the prodromal phase [7,18,19]. Interpretations of previous associations between PD and these diagnoses, particularly epilepsy, have been cautious due to the confounding effects of AEDs such as sodium valproate. However, our analysis suggests that epilepsy and AEDs each contribute to the risk factor of incident PD.

The strengths of this study are its inclusion of more than 24,000 PD patients and examination of a number of different AEDs in a primary care population. Furthermore, we were able to adjust for the presence of underlying diagnoses that have been reported to be associated with incident PD. However, the study is also subject to several limitations. First, as we used electronic medical records from primary care practices, we did not have information on how the PD cases were diagnosed (i.e., diagnostic criteria or assessment tools). The diagnosis of PD relied on ICD-10 codes alone, and no data were available on symptoms (e.g., tremor, bradykinesia, muscle stiffness, and others). Second, both epilepsy and PD may have been diagnosed and treated in specialized practices and hospitals, and some of the related data could have been undocumented in the electronic medical records of general practitioners in the Disease Analyzer database. Third, no data were available on smoking status and alcohol use that would allow these variables to be taken into consideration as PD risk factors. 

## 5. Conclusions

This retrospective study including adults followed in general practices in Germany found that not only epilepsy, restless legs syndrome, and neuropathy but also AEDs including sodium valproate, gabapentin, and pregabalin were associated with an increased risk of subsequent PD. Further studies on these associations among older adults, especially investigations conducted in other countries, are needed to confirm the present results.

## Figures and Tables

**Table 1:** Demographic and clinical characteristics of the study sample (after 1:1 matching).

Variable	PD Cases (*n* = 24,950)	Non-PD Controls (*n* = 24,950)	*p*-Value ^1^
Mean age (standard deviation)	75.2 (10.5)	75.2 (10.5)	1.000
Age ≤ 60 (%)	9.3	9.3	1.000
Age 61–70 (%)	15.7	15.7	
Age 71–80 (%)	41.7	41.7	
Age > 80 (%)	33.3	33.3	
Female (%)	47.3	47.3	1.000
Male (%)	52.7	52.7	
Pre-diagnostic observation time in years, mean (standard deviation)	7.8 (5.0)	7.8 (5.0)	1.000

Data are given as percentages unless otherwise stated. PD = Parkinson’s disease. ^1^ *p*-values were obtained using Wilcoxon signed-rank tests for continuous age and the average number of medical consultations per year during the follow-up time, the Stuart–Maxwell test for categorical age, and McNemar tests for comorbidities.

**Table 2:** Association between antiepileptic drugs and incident Parkinson’s disease among patients followed in German primary care practices.

AED Drug	Cases (%)	Controls (%)	COR for PD (95% CI)	*p*-Value	AOR for PD (95% CI)	*p*-Value
Any AED	12.1	6.5	2.00 (1.87–2.13)	<0.001	1.43 (1.33–1.53)	<0.001
Gabapentin	4.3	2.2	1.96 (1.76–2.17)	<0.001	1.36 (1.22–1.52)	<0.001
Pregabalin	5.2	2.9	1.87 (1.70–2.04)	<0.001	1.28 (1.15–1.41)	<0.001
Carbamazepine	1.3	0.9	1.46 (1.23–1.83)	<0.001	0.88 (0.73–1.06)	0.169
Sodium valproate	1.2	0.3	3.78 (2.95–4.85)	<0.001	2.39 (1.84–311)	<0.001
Levetiracetam	1.1	0.6	1.79 (1.46–2.19)	<0.001	1.03 (0.83–1.29)	0.803
Lamotrigine	0.4	0.2	1.89 (1.35–2.64)	<0.001	1.05 (0.73–1.50)	0.804
Epilepsy	4.2	1.8	2.39 (1.14–2.67)	<0.001	1.91 (1.69–2.15)	<0.001
Restless legs syndrome	7.2	2.1	3.62 (3.28–4.00)	<0.001	3.02 (2.73–3.34)	<0.001
Neuropathy	14.2	8.2	1.84 (1.74–1.95)	<0.001	1.53 (1.44–1.62)	<0.001

COR: Crude odds ratio; AOR: Adjusted odds ratio. All multivariable analyses were adjusted for diagnoses of epilepsy, restless legs syndrome, and neuropathy as well as other AEDs.

**Table 3:** Association between antiepileptic drugs prescribed in the past and incident Parkinson’s disease among patients followed in German primary care practices.

AED Drug	AEDs Prescribed at Least 12 Months Prior to Index Date		AEDs Prescribed at Least 36 Months Prior to Index Date	
	AOR for PD (95% CI)	*p*-Value	AOR for PD (95% CI)	*p*-Value
Any AED	1.50 (1.39–1.62)	<0.001	1.43 (1.26–1.63)	<0.001
Gabapentin	1.40 (1.24–1.60)	<0.001	1.41 (1.14–1.74)	0.002
Pregabalin	1.49 (1.33–1.67)	<0.001	1.98 (1.54–2.53)	<0.001
Carbamazepine	0.89 (0.73–1.07)	0.214	0.80 (0.62–1.03)	0.082
Sodium valproate	2.14 (1.60–2.87)	<0.001	1.11 (0.70–1.75)	0.663
Levetiracetam	0.92 (0.70–1.20)	0.530	2.00 (0.80–4.96)	0.137
Lamotrigine	1.33 (0.87–2.03)	0.196	1.21 (0.51–2.86)	0.668
Epilepsy	1.99 (1.77–2.23)	<0.001	2.21 (1.97–2.47)	<0.001
Restless legs syndrome	3.20 (2.90–3.54)	<0.001	3.27 (2.96–3.62)	<0.001
Neuropathy	1.58 (1.49–1.68)	<0.001	1.65 (1.55–1.75)	<0.001

AOR, Adjusted odds ratio. All multivariable analyses were adjusted for diagnoses of epilepsy, restless legs syndrome, and neuropathy and other AEDs.

## Data Availability

The data that support the findings of this study are available from the corresponding author on reasonable request.
